# The Evolution of Epigenetic Regulators *CTCF* and *BORIS/CTCFL* in Amniotes

**DOI:** 10.1371/journal.pgen.1000169

**Published:** 2008-08-29

**Authors:** Timothy A. Hore, Janine E. Deakin, Jennifer A. Marshall Graves

**Affiliations:** ARC Centre for Kangaroo Genomics, Research School of Biological Sciences, The Australian National University, Canberra, Australian Capital Territory, Australia; University of Cambridge, United Kingdom

## Abstract

CTCF is an essential, ubiquitously expressed DNA-binding protein responsible for insulator function, nuclear architecture, and transcriptional control within vertebrates. The gene *CTCF* was proposed to have duplicated in early mammals, giving rise to a paralogue called “brother of regulator of imprinted sites” (*BORIS* or *CTCFL*) with DNA binding capabilities similar to *CTCF*, but testis-specific expression in humans and mice. *CTCF* and *BORIS* have opposite regulatory effects on human cancer-testis genes, the anti-apoptotic *BAG1* gene, the insulin-like growth factor 2/H19 imprint control region (*IGF2/H19* ICR), and show mutually exclusive expression in humans and mice, suggesting that they are antagonistic epigenetic regulators. We discovered orthologues of *BORIS* in at least two reptilian species and found traces of its sequence in the chicken genome, implying that the duplication giving rise to *BORIS* occurred much earlier than previously thought. We analysed the expression of *CTCF* and *BORIS* in a range of amniotes by conventional and quantitative PCR. *BORIS*, as well as *CTCF*, was found widely expressed in monotremes (platypus) and reptiles (bearded dragon), suggesting redundancy or cooperation between these genes in a common amniote ancestor. However, we discovered that *BORIS* expression was gonad-specific in marsupials (tammar wallaby) and eutherians (cattle), implying that a functional change occurred in *BORIS* during the early evolution of therian mammals. Since therians show imprinting of *IGF2* but other vertebrate taxa do not, we speculate that *CTCF* and *BORIS* evolved specialised functions along with the evolution of imprinting at this and other loci, coinciding with the restriction of *BORIS* expression to the germline and potential antagonism with *CTCF*.

## Author Summary

Epigenetic mechanisms heritably change gene expression without altering DNA sequence. Currently, little is known about the evolution of epigenetic traits, and the genes that control them. *CTCF* is an essential epigenetic regulator that is expressed widely in the tissues of vertebrates and modifies the transcription of genes by altering their location within the nucleus. *CTCF* duplicated at some time during vertebrate evolution, giving rise to a similar gene called *BORIS* with expression that is limited to parts of the testes of humans and mice, but whose function is largely unknown. *BORIS* may contribute to the regulation of genomic imprinting, a form of epigenetic control specific to live-bearing mammals. We discovered *BORIS* in all mammal groups and reptiles, implying that its genesis from *CTCF* occurred much earlier than previously thought, preceding genomic imprinting by over 100 million years. *CTCF* and *BORIS* have not previously been found expressed together except in tumours, leading to the hypothesis that *CTCF* and *BORIS* have conflicting functions that cause cancer when allowed to overlap. We found that *CTCF* and *BORIS* are expressed alongside each other in multiple somatic and reproductive tissues of a reptile (bearded dragon) and an egg-laying mammal (platypus), but that *BORIS* is restricted to the gonads of live-bearing mammals (cattle and wallaby). This indicates that *BORIS* specialised during mammalian evolution, in concert with the evolution of genomic imprinting.

## Introduction

CCCTC-binding factor (CTCF) is a ubiquitously expressed protein that binds to more than 20,000 sites within the human genome [1]–[3]. The distribution of these binding sites, along with experimental data from several well-characterised loci (reviewed [4]) indicates that CTCF acts as an insulator protein genome-wide, defining boundaries for gene clusters or segregating alternative promoters. This can affect gene expression, for instance at the well-studied chicken *ß–globin* locus, where CTCF binding to the FII insulator leads to transcriptional silencing by blocking the effects of a nearby enhancer [5].

CTCF is also required for inter-chromosomal interactions such as pairing of the X chromosomes during initiation of X chromosome inactivation [6] and even co-localisation of non-homologous chromosomes [7]. It is now considered that CTCF contributes more broadly to the establishment of nuclear compartments where transcription is enhanced or repressed [8],[9], rather than functioning only to insulate neighbouring regions of the genome from each other. Given these diverse and significant roles, it is not surprising that CTCF is essential for life (reviewed [9]). Furthermore, point mutation and loss of heterozygosity of *CTCF* is associated with human cancer, identifying *CTCF* as an important candidate tumour-suppressor gene [10].

**Figure 1:**
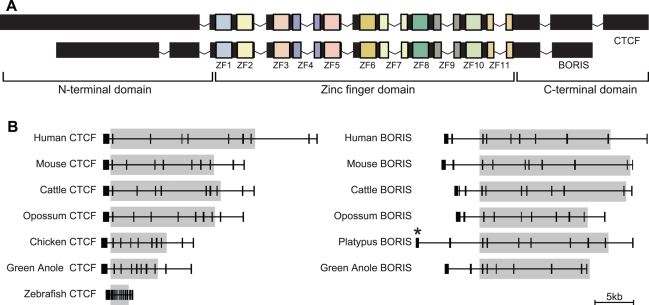
Gene structure of *CTCF* and *BORIS*. (A) *CTCF* and *BORIS* share a similar ZF domain, but different N- and C-terminal domains. (B) All vertebrate *CTCF* orthologues posses ten exons. Intron-exon boundaries are identical between all *CTCF* and *BORIS* orthologues within the ZF domain (grey). Note, genomic coverage of platypus *BORIS* is incomplete at the 5′ end (*).

The CTCF protein, and the nucleotide sequence that encodes it, can conceptually be divided into three separate domains (Figure 1A). The central (ZF) domain contains ten Cys_2_His_2_ zinc-fingers (ZFs), and one Cys_2_HisCys ZF, combinations of which are used to bind various DNA sequences [11]. Flanking the ZF domain are the N- and C-terminal domains, which interact with other DNA-binding proteins, histones and histone modifying proteins, and the large subunit of polymerase II (reviewed [8]). In all three of its domains, CTCF shows extraordinary conservation throughout vertebrates [11]–[14], and even non-vertebrates [15], reflecting the considerable functional constraint CTCF must face due to its multiple essential roles and many interacting partners.

In humans and mice, a paralogue of *CTCF* has been identified known as CTCF-like (*CTCFL*), or as it was originally named (and how we will refer to it hereafter), Brother Of Regulator of Imprinted Sites (*BORIS*) [16]. Human and mouse BORIS posses a suite of ZFs with binding capability, sequence and underlying gene structure that is extremely similar to CTCF (Figure 1A). However, the N- and C-terminal domains of human and mouse BORIS show almost no similarity to CTCF, implying that although they can bind the same DNA, they are likely to act differently at these sites.

One example of how CTCF and BORIS may function differently comes from their effects on the regulation of genomic imprinting, which is responsible for parent-of-origin specific, mono-allelic gene expression in about 100 mammalian genes [17]. The most extensively studied imprinted gene, insulin-like growth factor 2 (*IGF2*), is expressed exclusively from the paternally-derived chromosome in eutherian (‘placental’) mammals [18]–[21] and marsupial mammals [22],[23]. Located downstream of *IGF2* is the untranslated RNA *H19*, which is expressed solely from the maternally derived chromosome [24],[25]. Biallelic expression of *IGF2* was discovered in the egg-laying monotreme mammals [26], birds [22],[27] and fish [28], implying that imprinting of this region evolved at the same time as viviparity, 180-210MYA [29].

In mice, imprinted expression of *Igf2/H19* depends on the imprint control region (ICR), an insulator element located between these two genes. The ICR is methylated during spermatogenesis, specifically marking the paternally-derived chromosome [30]. CTCF binds to the ICR, but only on the unmethylated, maternally-derived chromosome. When bound to the maternally-derived ICR, CTCF performs many functions including protecting the ICR from methylation [31]–[33], blocking *Igf2* access to a downstream enhancer (resulting in *Igf2* silencing *in cis* [34],[35]), and simultaneously activating *H19* expression [33]. CTCF is thought to orchestrate these events through the formation of maternal-specific chromosomal loops [36] and the establishment of local chromatin modifications [37]. Thus, CTCF acts somatically to ‘*interpret*’ the differential methylation mark of the ICR acquired during gametogenesis, resulting in imprinted expression of *Igf2/H19*.

In contrast, BORIS appears to be essential for the *establishment* of differential methylation at the *IGF2/H19* ICR [38]. In mouse testes, BORIS is bound to the *Igf2/H19* ICR during the time when the ICR becomes methylated. Methylation is accomplished by members of the *de novo* methyltransferase 3 family, of which DNMT3L is essential to this process [39],[40] and DNMT3A/3B are partially redundant [41]. Transgenes containing the mouse ICR were methylated in *Xenopus* oocytes only when co-injected with BORIS, DNMT3L, one of DNMT3A/3B and a histone modifier called protein arginine methyltransferase 7 (PRMT7) [38]. Thus, BORIS and CTCF both bind to the ICR through their common ZF domain, yet appear to act differently at this site. BORIS *establishes* differential methylation of the ICR and later CTCF *interprets* this mark, resulting in imprinted expression of *Igf2/H19*.

Significantly, in humans and mice *CTCF* and *BORIS* show mutually exclusive expression; *BORIS* is transcribed only in certain parts of the developing and adult testes, whereas *CTCF* is expressed in all other regions tested [16],[38]. The only reported instances of *BORIS* expression outside of the testes is in various types of cancers [42]–[47]. This mutually exclusive expression pattern could be explained in part by the recent discovery that CTCF actually binds to the promoter of *BORIS* and negatively regulates its expression [44].

*BORIS* is associated with a large group of potentially oncogenic “cancer-testis” (CT) genes, which also show testis-specific, or gonad-specific, expression in healthy individuals, but are highly expressed in cancers [48]. CTCF binds to the promoter of many CT-genes in healthy somatic tissue where these genes are silenced [42],[43],[49],[50]. However, this repression is disrupted by conditional expression of BORIS, which replaces CTCF binding at the promoter and subsequently causes local demethylation and gene activation [42],[43],[49]. Similarly, CTCF-binding has a repressive effect on the promoter of the anti-apoptotic gene *BAG1*, whereas BORIS performs oppositely, altering histone methylation and upregulating *BAG1* expression [51]. The discovery that *CTCF* and *BORIS* have opposite effects on transcription of *BAG1*, some CT-genes, and on the epigenetic status of the *IGF2/H19* ICR, has lead to the (albeit controversial [47]) hypothesis that CTCF and BORIS are antagonistic regulators of the common loci to which they bind, and that inappropriate interactions between them is cancer promoting [16],[52].

Comparisons between the genomes of mammals and other vertebrates are powerful tools in understanding how human genes and their products are regulated, what their function is and how and why they evolved [53],[54]. Indeed, much of CTCF function has been characterised in chicken, including its capacity as an insulator protein [5] and recent studies have revealed the extreme conservation of *CTCF* sequence and function in amphibians [13], fish [14] and even invertertebrates such as *Drosophila* [15].

Despite this, *CTCF* has not been characterised in non-eutherian mammals or reptiles and whether *BORIS* exists outside humans and mice is not even known. From reported failures to find *BORIS* sequence in chicken and fish [14],[16] it has been proposed that *BORIS* arose recently from duplication of *CTCF* in an early mammal [16]. However, here we report that *BORIS* orthologues are present in all major mammalian groups and at least two reptilian species, proving that *BORIS* evolution occurred much earlier than has been recognised. We examined the expression pattern of *CTCF* and *BORIS* in the three major mammalian clades and a reptile, discovering that although *CTCF* is ubiquitously expressed in all species, *BORIS* became progressively specialised to testis throughout amniote evolution. We consider these new data with respect to current theories regarding *CTCF* and *BORIS* as antagonistic epigenetic regulators and their roles in governing genomic imprinting at the *IGF2/H19* locus.

## Results

We isolated, sequenced and characterised *CTCF* and *BORIS* homologues in eutherians, marsupials, monotremes and reptiles, and studied their expression profiles in one species from each of these vertebrate groups.

### Cloning and Characterisation of *CTCF* and *BORIS* Orthologues in Vertebrates

Homologues of *CTCF* and *BORIS* were amplified from a range of amniotes by reverse-transcriptase PCR (RT-PCR) and rapid amplification of cDNA ends, using primers designed from sequenced genomic data or evolutionarily conserved regions (Table S1). Full-length or near full-length protein coding cDNA sequences were retrieved in this way from our model eutherian, marsupial, monotreme and reptilian species; domestic cattle (*Bos taurus*), tammar wallaby (*Macropus eugenii*), duck-billed platypus (*Ornithorhynchus anatinus*) and central bearded dragon (*Pogona vitticeps*) respectively (accession numbers EU527852-EU527858). Similarity searches, using these sequences and other annotated *CTCF* and *BORIS* sequences as queries, were conducted in a variety of databases hosted at NCBI (http://www.ncbi.nlm.nih.gov) and Ensembl (http://www.ensembl.org). This approach identified a further 37 homologues of these genes in vertebrates (Table S2).

**Figure 2:**
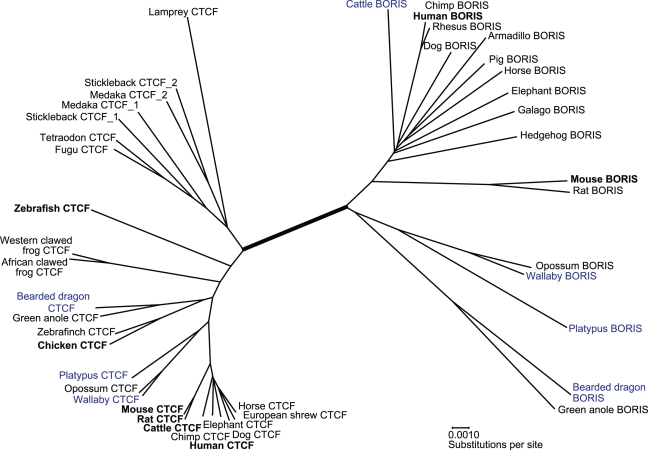
Neighbour-joining tree showing relationships between members of the *CTCF* and *BORIS* gene family. Sequence we determined experimentally (blue) and discovered by *in silico* similarity searches (black) form two distinct clusters with previously annotated *CTCF* and *BORIS* orthologues (bold). These clusters are separated from each other by a branch with 100% bootstrap value (thick line). Accession numbers and scientific names for these sequences and species are shown in Table S2.

From the largest region of common overlap between these homologues, a neighbour joining tree was constructed, revealing two distinct clusters of sequence (Figure 2). One of these clusters contained previously annotated copies of *CTCF* from human (NM_006565), mouse (NM_007794), rat (NM_031824), cattle (NM_001075748), chicken (NM_205332) and zebrafish (NM_001001844). The other cluster contained annotated *BORIS* sequence from human (NM_080618) and mouse (NM_001081387). The branch separating these two clusters was supported by a 100% bootstrap value. This unambiguously defined which sequences were *CTCF* orthologues and which were *BORIS* orthologues.

In line with previous studies, we detected *CTCF* orthologues in all major vertebrate groups [12]–[14]. Included in this cluster were closely related duplicate *CTCF* sequences (designated *CTCF*1 and *CTCF*2) from stickleback and medaka. The *BORIS* cluster included, as well as orthologues from many eutherian species, clear orthologues in two marsupials (Gray short-tailed opossum, *Monodelphis domestica*, and wallaby), a monotreme (platypus) and two reptiles (bearded dragon and green anole, *Anolis carolinensis*). No orthologues of *BORIS* could be detected using nucleotide BLAST, or translated BLAST searches in genomes of any avian (chicken, *Gallus gallus*; and zebra finch, *Taeniopygia guttata*), amphibian (Western-clawed frog, *Xenopus tropicalis*), teleost fish (puffer fish, *Takifugu rubripes* and *Tetraodon nigroviridis*; zebrafish, *Danio rerio*; stickleback, *Gasterosteus aculeatus*; and medaka *Oryzias latipes*) or primitive vertebrate (sea lamprey, *Petromyzon marinus*).

Although BLAST searches failed to identify any sequence orthologous to *BORIS* in bird, amphibian and fish genomes, it remained possible that *BORIS* is present in these genomes but is too diverged to detect using standard alignment methods. This seemed particularly likely for chicken, as birds are a sister taxon to the reptiles, in which we discovered *BORIS* orthologues. Applying a strategy previously used in the search for divergent genes [55], we sought orthologues of markers on either side of human *BORIS.* We located such sequences in multiple species, and searched the dividing spaces for *BORIS*-like sequence.

We found that genes flanking *BORIS* in humans were part of a single large block of genes (*TMEPAI-BMP7*) clustered together in the same orientation in all tetrapods (data not shown). Genes from this block were either not clustered together, or were not present in sea lamprey and teleost fish genomes.

**Figure 3:**
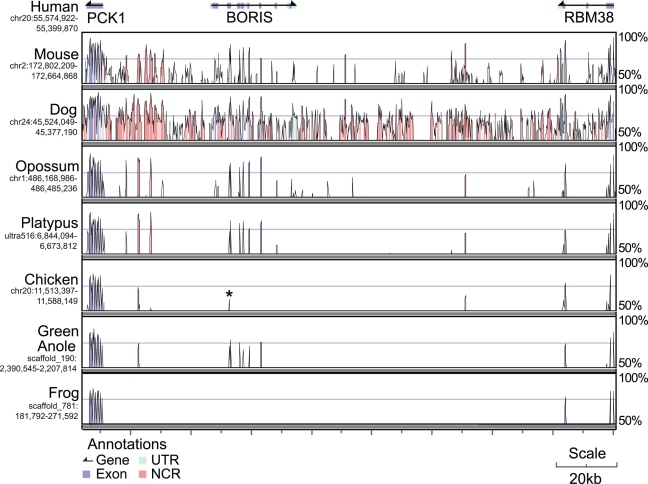
Human genomic sequence encompassing *PCK1-BORIS-RBM38* (top) compared to the orthologous regions in other amniotes. High similarity over a 100bp window is seen for most exonic sequence (blue) and some untranslated regions (UTR, light blue) or non coding regions (NCR, pink). Despite no similarity to any other region of human *BORIS* to the chicken *PCK1-RBM38* region, the peak labelled with a star is homologous to the first ZF of *BORIS*.

We aligned the regions containing genes immediately adjacent to *BORIS* (*PCK1* and *RBM38*) between human, mouse, dog, opossum, platypus, chicken, green anole and frog (Figure 3). As before, we could detect no *BORIS* orthologues in frog, but found some similarity between the first zinc finger of *BORIS* and a 108-bp region of the chicken *PCK1-RBM38* intergenic sequence. When this sequence was used a query for reciprocal BLAST against the entire human genome, the best alignments were to the first zinc finger of *CTCF* and *BORIS*, indicating that these sequences were homologous. We could uncover no evidence for this sequence being part of an active gene other than finding that it overlaps with an Ensembl *ab-initio* gene prediction (GENSCAN00000030237). We therefore conclude that the sequence is a degraded relic of *BORIS*.

### Sequence Analysis and Gene Structure of *CTCF* and *BORIS* in Vertebrates

We aligned predicted CTCF and BORIS proteins and found that, like previously annotated versions of these proteins, all possessed eleven ZFs, ten of which belong to the Cys_2_His_2_ class, and one which belongs to the Cys_2_HisCys class (Figure S1). As previously reported for human, mouse, chicken, zebrafish and frog [12]–[14],[16], we found that all vertebrate CTCF orthologues are extremely highly conserved throughout their entire length. From pairwise alignments over the entire length of its sequence, we found 92% average identity between human CTCF and other selected vertebrate CTCF sequences (Table 1). In comparison, similarity of BORIS orthologues, to each other and to CTCF, was largely restricted to the region encoding the ZFs. When human BORIS was compared to other BORIS sequences the average identity was 80.4% within the ZF domain, but less than 35% similar in the other two regions. Moreover, comparisons of human BORIS with CTCF sequences produced an average identity of 74.1% within the ZF domain, but less than 15% conservation within the other regions.

**Table 1:** Average pairwise similarity (%) between regions of human CTCF/BORIS and other vertebrate orthologues.

		Human CTCF				Human BORIS			
		N-term	ZF	C-term	Total	N-term	ZF	C-term	Total
**Average pairwise identity with vertebrate**	**CTCF orthologues**	90.1	99.5	80.7	92.0	14.2	74.1	10.2	38.0
	**BORIS orthologues**	9.7	72.8	8.6	35.8	32.3	80.4	23.7	53.1

Note, as not all sequences we discovered (Table S2) were of ideal length for pairwise comparisons, identities were calculated using human, mouse, dog, cattle, elephant, opossum, wallaby, platypus, chicken, bearded dragon, green anole, frog (*X. tropicalis* and *X. laevis*) and zebrafish sequences.

*CTCF* genomic sequence from human, mouse, zebrafish and frog were all reported to have ten protein-encoding exons when they were first characterised [13],[14],[16]. In contrast, chicken *CTCF* was reported by Klenova *et al*. [12] to only have seven protein coding exons, four of which contained all eleven zinc fingers. We analysed the gene structure of *CTCF* in all species from which there was full genomic sequence and found that all sequences, including chicken *CTCF*, contained ten exons in total, with seven ZF exons (Figure 1B). *BORIS* orthologues were also found to have a very similar structure, especially within the ZF domain where intron-exon boundaries were identical.

### Gene Expression Analysis

One of the most remarkable characteristics of *CTCF* and *BORIS* is that in humans and mice they show apparently mutually exclusive expression. *BORIS* is transcribed only in specific parts of the testis, while *CTCF* is expressed in all tissues except those expressing *BORIS* [16],[38]. This expression pattern underpins the hypothesis that *BORIS* is the key regulator establishing the male germline imprint of *IGF2*, that it acts antagonistically to *CTCF* and defines its inclusion within the cancer-testis group of genes.

**Figure 4:**
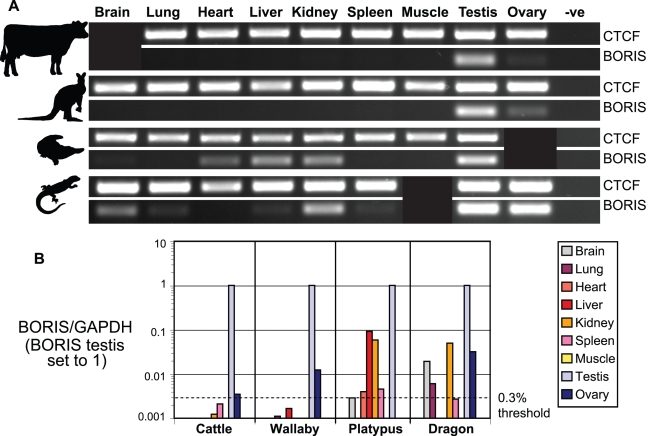
Expression analysis of *CTCF* and *BORIS*. (A) Conventional RT-PCR of *CTCF* and *BORIS* after 35 cycles. Note, cattle brain, platypus ovary and bearded dragon muscle could not be tested due to tissue unavailability or poor RNA quality. (B) *BORIS* transcript levels relative to the positive control gene *GAPDH* as quantified by real-time PCR. To assist comparisons between species, we set *BORIS* expression in the testis to 1 and adjusted all other values within the same species proportionally. As found in humans, expression of *BORIS* in somatic tissues of cattle and wallaby did not exceed 0.3% of that found in testis, although ovarian expression is just on or above this threshold. In contrast, levels of *BORIS* expression in platypus (liver and kidney) and bearded dragon (brain, kidney and ovary) are well above this threshold and in some cases match *CTCF* expression (Figure S2).

To determine if this expression pattern is conserved more widely in vertebrates, we examined the transcription of *CTCF* and *BORIS* in cattle, wallaby, platypus and bearded dragon. Initially, we performed 35 cycles of RT-PCR on a series of tissues using *CTCF/BORIS* primers anchored within at least one of the ZFs and a surrounding non-zinc finger region (Table S1). *CTCF* transcripts were detected in this way for all tissues and animals tested (Figure 4A). *BORIS* transcripts were detected only in the gonads of cattle and wallaby; strongly in testes, and weakly in ovarian samples. In contrast, *BORIS* was amplified from a much wider set of somatic and reproductive tissues in platypus (brain, heart, liver, kidney and testis); and bearded dragon (brain, lung, liver, kidney, spleen, testis and ovary). To minimise the possibility we were observing tissue specific splice variants, we repeated our RT-PCR experiments using primers from different regions of *BORIS* (Table S1), and found similar results (data not shown).

Despite these discoveries, due to the nature of conventional ‘end-point observed’ RT-PCR our initial experiments were semi-quantitative at best. Thus, we were unsure if the expression we were observing was at a level which was biologically relevant. A recent publication using the quantitative real-time PCR technique found that although *BORIS* expression is considered to be restricted to the testis and some tumours, *BORIS* transcripts could be detected in other tissues up to 0.3% of the level of *BORIS* in the testis [47]. The authors concluded from this that expression of *BORIS* less than 0.3% of the level in testis was not biologically relevant.

We performed real-time PCR amplifications of *CTCF* and *BORIS* on all our available tissues in triplicate and comparatively quantified their respective levels using the Corbett Research Rotorgene system, with SYBR Green as the fluorescent DNA-binding dye. Differences in template concentration within a species were taken into consideration by normalising our results to the housekeeping gene glyceraldehyde-3-phosphate dehydrogenase (*GAPDH*). In agreement with our initial RT-PCR experiments, amplification of *CTCF* occurred in all tissues and species reproducibly, but with up to 50-fold variation between tissues (Figure S2) not unlike that seen previously in developing zebrafish and frog [13],[14].

Like previous experiments, we found that *BORIS* amplifications by real-time PCR were predominantly from the testis, with consistently high expression between 10% and 100% of the level of *GAPDH* (Figure S2). As expected, *BORIS* amplification was also detected on multiple occasions outside of the testis, particularly within platypus and bearded dragon, and at levels well within the expected limitations of our assay (see methods). When we applied the 0.3% cut-off defined by Kholmanskikh *et al*., [47] to our results, we found that as in humans, levels of *BORIS* in somatic tissues were below this threshold in cattle and wallaby (<0.2% of testis expression), while ovarian *BORIS* levels were just on (cattle) or above (wallaby) this threshold (Figure 4B).

Expression of *BORIS* outside of the testis in platypus and bearded dragon was much higher. *BORIS* transcripts in the liver and kidney of platypus was within 6–10% of that found in platypus testis (Figure 4B), and was at a level comparable to *CTCF* expression found in these tissues (Figure S2). Likewise, levels of *BORIS* transcripts in bearded dragon brain, kidney and ovary were 2–5% of the level of *BORIS* in testis. *BORIS* transcripts were detected in other five other somatic tissues of platypus (brain, heart and spleen) and bearded dragon (lung and spleen) at levels just on or above the 0.3% threshold.

## Discussion

We examined the sequence and expression of key epigenetic regulators *CTCF* and *BORIS* in vertebrates through cloning, sequencing, bioinformatic analysis and quantitative gene expression experiments. Our results are at variance with the hypothesis that *BORIS* arose recently by duplication of *CTCF* in mammals, and was quickly specialised for a role in germ cell imprinting that was complementary, or even antagonistic, to the role of *CTCF*.

### *BORIS* First Arose in Early Amniotes

Previous studies established that *CTCF* is a highly conserved and ubiquitous gene in humans and mice, as well as other vertebrates including birds, fish and amphibians [12]–[14]. Our studies on cattle, wallaby, platypus and dragon lizard confirm the expectation that *CTCF* is highly conserved in all vertebrate groups, and is expressed to varying degrees in all tissues of eutherian, marsupial and monotreme mammals, as well as reptiles (Figures 4A and S2).

In contrast to the well-studied *CTCF* gene, much less is known about the evolutionary history and function of *BORIS*. *BORIS* sequence was previously determined only in humans and mice [16], and no orthologue was detected in chicken. This gave rise to the speculation that *BORIS* duplicated from *CTCF* only recently in the mammal lineage. In addition, chicken *CTCF* was reported to have a gene structure significantly different from that of mammal *CTCF* and *BORIS*. Chicken was therefore considered to represent the ancestral gene structure, and an alteration of *CTCF* gene structure was proposed to have occurred in the mammalian ancestor, followed by a duplication to give rise to *BORIS*.

We found that chicken *CTCF* was not, after all, different in structure from mammal *CTCF* as was previously reported [12],[16] (Figure 1B). The chicken genome project had not been undertaken when chicken *CTCF* was initially sequenced, so sequence coverage from this early study may not have been sufficient to build a reliable assembly of the region. Alternatively, the *CTCF* clone that was sequenced may have been a cDNA and genomic DNA chimaera.

**Figure 5:**
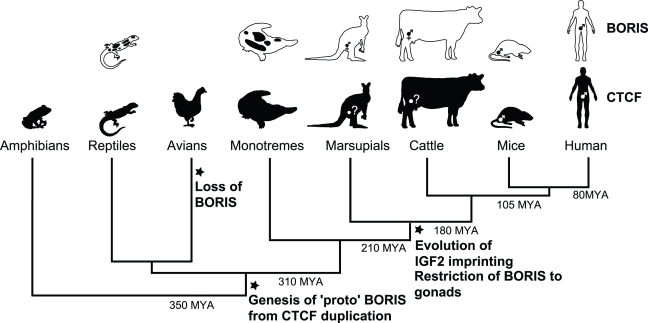
Proposed model of *CTCF* and *BORIS* evolution in amniotes. The expression of *CTCF* and *BORIS* is indicated (black = expressed, white = not expressed) within various taxa. The ancestral expression pattern of *BORIS* is wide, including multiple somatic tissues (reptiles and monotremes), but becomes progressively restricted in therian mammals with gonad specific expression in marsupials and cattle (Figure 4) and testis-specific expression in humans and mice [16],[38],[47]. Significant events in the evolution of *CTCF* and *BORIS* are marked with respect to the phylogenetic tree.

Unexpectedly, we found orthologues of *BORIS* in at least two reptilian species (bearded dragon and green anole). This means that the duplication of *CTCF* which gave rise to *BORIS* must have occurred prior to the divergence of sauropsids (birds and reptiles) and mammals 210–310 million years ago (Figure 5). In agreement with Loukinov *et al*. [16] we could find no full orthologue of *BORIS* within the chicken genome. However, by analysing the intergenic region between markers flanking the expected site of *BORIS* in chicken, we did discover a small 108-bp segment of DNA homologous to the first zinc finger of *BORIS* (Figure 3). Although this region of DNA may be part of another functional gene, we consider that it is unlikely to be functionally related to other *BORIS* orthologues, given that no other regions showed conservation, even the usually well-conserved zinc fingers. We conclude that either *BORIS* succumbed to pseudogenisation in birds some time after they diverged from reptiles, or underwent a rapid functional change leaving behind only small traces of its evolutionary past.

In an extension of previous studies [16], we found extremely high conservation between vertebrate CTCF orthologues, but observed that BORIS homologues were similar to each other, and to CTCF, only within the ZF domains (Table 1). These observations support the prediction of Loukinov *et al*. that any major differences between CTCF and BORIS function are probably attributable to the N- and C-terminal domains, given these are the most divergent. In fact, the N- and C-terminal domains of CTCF and BORIS contained only small pockets of sequence that were obviously alignable (Figure S1). Interestingly, two of these conserved regions overlapped the start and end of these proteins, implying that the duplication that gave rise to *BORIS* must have involved the entire *CTCF* sequence.

The ZF domain of CTCF orthologues we examined showed an almost perfect (99.5% average) identity with human CTCF. In comparison, the average conservation between the ZF domain of human BORIS and other BORIS orthologues was much lower (80.4%). This suggests that BORIS experienced a decrease in functional constraint relative to CTCF, initially because it was a duplicate, and presently because it only binds a subset of the sites bound by CTCF. Alternatively, BORIS may bind some sequences not recognised by CTCF [38]. In support of this, we found that although all of the amino acids thought to perform protein-DNA interactions [56] were 100% conserved for vertebrate CTCF, many were not conserved in some, or all BORIS orthologues (Figure S1). It would be interesting to investigate this further by mapping BORIS binding sites in the genome relative to the published CTCF binding sites [2],[3].

### Expression of Ancestral *BORIS* in Somatic Tissue

Our RT-PCR experiments showed two main patterns of *BORIS* expression (Figure 4). In the marsupial and the eutherian (wallaby and cattle respectively) we found predominantly testis-specific expression with some ovarian expression, whereas in the reptile (bearded dragon) and the monotreme (platypus) we detected expression of *BORIS* in multiple somatic tissues as well as the gonads. When these experiments were repeated using quantitative real-time PCR, we discovered that after 45 cycles of PCR, some *BORIS* transcripts could be detected outside of the germline in cattle and wallaby. However, we found that these levels of *BORIS* were extremely low, approaching the limits of detection and falling under a previously defined threshold for meaningful expression of *BORIS* [47]. Thus, we expect that *BORIS* function in cattle and wallaby is absent from somatic tissues, just as is predicted in humans and mice. More experiments will be required to determine if the ovarian expression of *BORIS* in cattle and particularly wallaby is functionally significant.

The highest levels of *BORIS* expression outside of the testis were found in platypus and bearded dragon. The most striking examples of these came from the liver and kidney of platypus and the brain, kidney and ovary of bearded dragon, which were at levels 2-10% of *BORIS* expression in the testis (Figure 4B). In two cases (platypus liver and kidney) this level of expression was close to the level of *CTCF* expression within the same tissues (Figure S2). These results strongly suggest that *BORIS* in platypus and bearded dragon functions outside of the testes, including in the ovary and multiple somatic tissues. This finding is of significance because it indicates that *BORIS* had wide expression in an ancestral amniote, similar to that of *CTCF,* the gene from which it arose by duplication.

The question then arises, why was the *CTCF* duplicate (or “proto-*BORIS*”) initially retained and why did it succumb to evolutionary change? The high degree of *CTCF* conservation throughout vertebrates implies that it is a gene under extreme functional constraint. Accordingly, perhaps it is not surprising that a *CTCF* duplicate in a new genomic environment would be retained and undergo sub-functionalisation, alleviating some mutational load upon CTCF. Subfunctionalisation may also explain why duplicate copies of *CTCF* have been retained in the genomes of medaka and stickleback (Figure 2), following whole-genome duplication of early teleost fish [57].

Although we observed *CTCF* and *BORIS* expression alongside each other in some tissues of monotremes and reptiles, these genes are apparently not co-expressed in humans and mice and may even be antagonistic. CTCF and BORIS bind competitively to common sites and display opposing effects on the epigenetic status of the *Igf2/H19* ICR and transcription of *BAG1* and the CT-genes [16],[42],[43],[49],[52]. Thus, at some stage during the evolution of therian mammals, *CTCF* and *BORIS* evolved mutually exclusive expression and potential antagonism. As our studies were performed on whole tissues, we could not resolve whether *CTCF* and *BORIS* show mutually exclusive expression amongst the many discrete cell-types in testis and ovary in wallaby and cattle, so we cannot pinpoint when mutually exclusive expression arose in therian mammals after their divergence from the monotremes.

### *BORIS* Specialisation Correlates with the Evolution of Imprinting

To date, the only non-pathological function proposed for *BORIS* is the establishment of paternal-specific methylation at the *Igf2/H19* ICR in mice [16],[38]. If found to be true for mice, it seems likely that this function is conserved in humans, since they also possess a paternally methylated CTCF-dependent insulator (the ICR) [34],[35] and testis-specific *BORIS* expression which is exclusive of *CTCF* [16]. Moreover, differential methylation of CTCF/BORIS binding sites upstream of a maternally-expressed *H19* orthologue has been discovered in sheep and wallaby [58]-[60], suggesting that the mouse model of *Igf2/H19* imprinted regulation and *BORIS* function may be conserved throughout all therians. Yet, *BORIS* is not expected to have this function in reptiles and monotremes, or the amniotic ancestor from which *BORIS* first arose, as *IGF2* imprinting evolved after the divergence of monotremes from therian mammals. Our finding that *BORIS* expression is gonad-specific in wallaby and cattle, both of which possess imprinting of *IGF2* [21],[23], implies that restriction of *BORIS* expression to the germline correlates with the evolution of genomic imprinting at *IGF2/H19* and other loci (reviewed [61]).

In support of this, the evolution of another essential regulator of the *Igf2/H19* ICR is also strongly correlated with the evolution of imprinting. Orthologues of the *de novo* methyltransferase family member *DNMT3L* are present in eutherians and marsupials (which posses imprinting), but apparently not in chicken, fish [62] or platypus (T.H., unpublished data) which are thought to lack imprinting.

### Model of CTCF and BORIS Evolution in Amniotes

We propose that a duplication of *CTCF* occurred in a common ancestor of all amniotes, probably some time after their divergence from amphibians 350-310MYA (Figure 5). We predict that originally this ‘proto-*BORIS*’ functioned alongside *CTCF*, perhaps subfunctionalising to take on tissue-specific roles from the highly conserved and functionally constrained CTCF protein. When genomic imprinting arose in early therian mammals 210-180MYA, *BORIS* was recruited to perform imprint establishment in germ cells, and *CTCF* imprint interpretation at *IGF2/H19* and potentially other imprinted genes. We speculate that this specialisation marked the start of antagonism between *BORIS* and *CTCF*, through the development of opposing epigenetic effects at the common loci to which they bound. The result of this was restriction of *BORIS* expression to the gonads of early therian mammals, and later restriction to the testes in the ancestor of humans and mice.

The divergent nature and proposed clash of function between *CTCF* and *BORIS* has often been described as ‘sibling-rivalry’ [16],[52]. Our results show that this rivalry did not always exist, and ironically may have evolved in response to the evolution of genomic imprinting, which is in turn thought to have evolved from other conflicts in the family [63].

## Materials and Methods

### Tissue

Adult cattle tissue was sourced from commercial abattoirs processing farmed animals from New South Wales, Australia. Tissue from adult wallaby and platypus were sourced from a captive breeding colony of wallabies and a platypus tissue collection, both held at the Research School of Biological Sciences, Australian National University, Canberra, Australia. Juvenile central bearded dragon tissues samples were sourced from a captive breeding colony held at the University of Canberra, Australia. All tissue (excluding testes samples) was from females, except for platypus tissue which was male. The captivity and sacrifice of all animals was approved by the Australian National University (wallaby and platypus) and University of Canberra (bearded dragon) Animal Experimentation Ethics Committees (AEECP R.CG.08.03, R.CG.02.00 and CEAE 04/04 respectively). Sourcing of cattle tissue was exempt from AEEC approval, as these animals were not sacrificed primarily for research purposes (Simon Bain, ANU AEEC).

### Nucleic Acid Extraction, Amplification, and Sequencing

Genomic DNA extraction was performed on liver tissue samples following the standard protocol for mammalian tissue [64]. Total RNA was extracted using the GenElute Mammalian Total RNA Miniprep Kit (Sigma-Aldrich) according to the manufacturer's instructions. Eluted RNA was treated by DNAse digestion, using the DNA-free Dnase kit (Ambion) as recommended by the manufacturer. All samples were checked for quality and purity on a 1.2% denaturing formaldehyde agarose gel [64]. RNA was tested for genomic DNA contamination by PCR prior to first strand synthesis of cDNA. Approximately 800 ng of purified RNA was used to create cDNA using the SuperScript III Reverse Transcriptase system (Invitrogen) according to manufacturer's instructions. All first strand synthesis reactions were undertaken using random hexamer primers except for Rapid Amplification of cDNA Ends (RACE) experiments, where the GeneRacer Oligo dT primer (Invitrogen) was used. Conventional PCR amplifications were performed in a 50 µL reaction, including either 1 µL of undiluted cDNA or 200 ng of genomic DNA as a template, 0.2 µM of each primer (Table S1) and the following reagents from Invitrogen; 1X PCR Buffer, 0.8 mM dNTP mixture (0.2 mM each), 1.5 mM MgCl_2_ and 0.2 µL of Platinum Taq DNA Polymerase. Cycling conditions used were as follows; 94°C, 2 min; 34×(94°C, 30 sec; 61°C, 30 sec; 72°C, 1 min); 72°C, 10 min. When amplifications over 1000 bp were performed, extension times were increased by 1 min/kb. Nested PCR amplifications for 3′ RACE were also undertaken using this protocol, except with reduced cycle numbers, modified primer concentration and increased annealing temperatures as stipulated in the GeneRacer kit (Invitrogen) protocol.

For initial gene expression studies 5 µL of *CTCF* and *BORIS* amplified products were combined together with 6 µL of loading buffer (30% glycerol, with light Bromophenol blue staining) and subjected to electrophoresis for 40min at 7.6 V/cm on a 1% agarose gel with TAE buffer and SYBR Safe DNA gel stain (Invitrogen). Gels photographs were illuminated with blue light and exposed using the Gel Logic 100 Imaging System (Kodak). Other than cropping, no alterations to these images were performed.

Full length *CTCF* and *BORIS* cDNAs were amplified from liver and testes samples respectively and cloned using the TOPO TA Cloning Kit. Recombinant plasmid DNA was purified using the Wizard Plus SV Miniprep System and then combined with relevant primers (Table S1) for sequencing at the Australian Genome Research Facility.

### Real-Time PCR

Real-time PCR was performed in 20 µL reactions using the QuantiTect SYBR Green PCR Kit (Qiagen) according to manufacturer's instructions. Amplifications were performed and detected with a Rotorgene 3000 cycler (Corbett Research) using the following cycling conditions; 95°C, 15 min; 45×(94°C, 30 sec; 58°C, 30 sec; 72°C, 20 sec); 72°C, 10 min. All experimental amplifications were performed in triplicate and averaged over two or three concordant results which varied by C_t_ values of less than 0.7. Levels of *CTCF* and *BORIS* relative to *GAPDH* in each tissue and species were calculated using the comparative quantitation software supplied by Rotorgene. All products were checked for specificity by melt-curve analysis and electrophoresis.

Primers used in this analysis were designed for each species from similar intron-spanning regions of *CTCF*, *BORIS* and *GAPDH* (Table S1). These primers were selected for high amplification efficiency (>1.65) and low primer-dimer. A 10-fold serial dilution of testis cDNA was undertaken to determine the amplification range and performance of *BORIS* primers at low template concentrations, because *BORIS* (unlike *CTCF* and *GAPDH*) is known to have low or undetectable expression in many tissues [16],[47]. We found that *BORIS* transcripts could be detected reliably down to the 10^−3^ dilution. Primers for the positive control gene *GAPDH* (Table S1) were designed from sequence deposited on NCBI for cattle (NM_001034034.1), platypus (EH003224) and wallaby (EF654515 and trace archive data). For bearded dragon, *GAPDH* primers were designed from sequence we determined ourselves by PCR amplification and sequencing (EU784660).

### Bioinformatic Analysis

Homology searches were performed using BLASTn and tBLASTn [65] against the non-redundant, expressed sequence tag and trace archive databases at the NCBI website (http://www.ncbi.nlm.nih.gov) or release 46 of the Ensembl website (http://www.ensembl.org). For species in which gene prediction was not available, or was unrealistic, we performed our own gene predictions using Genomescan [66] and local alignment. A multiple alignment of the resulting set of predicted and experimentally determined cDNA sequence (Table S2) was produced using ClustalW2 with default parameters (http://www.ebi.ac.uk/Tools/clustalw2). Phylogenetic analysis was performed on aligned cDNA sequences by the neighbour-joining method with uncorrected distance measure, using the phylogenetic program PAUP* version 4.0 b 10 [67]. 1,000 replications were performed for bootstrap analysis. Protein coding predictions of these cDNA sequences were also made and aligned using ClustalW2. This alignment was then used to calculate pairwise identity between selected orthologues using MacVector v9.5.2.

The conserved block of genes orthologous to the region surrounding human *BORIS* was identified in amniote species by BLAST with the criteria of unique reciprocal best-hits back to the query sequence in the human genome. Genomic sequence from these orthologous blocks was extracted from Ensembl and aligned using the LAGAN algorithm [68] available on the mVISTA website with default parameters (http://genome.lbl.gov/vista/mvista/submit.shtml).

## Supporting Information

Figure S1 Alignment of predicted CTCF and BORIS sequence in a range of vertebrates. Amino acids from the 11 zinc fingers (blue), likely to interact with DNA are shown indicated with arrows, and regions of similarity at the start and end of CTCF and BORIS are highlighted in red. Note, this figure is not intended for printing. Rather, it should be viewed in a program such as Adobe Reader, which has zoom function.
(12.39 MB EPS)

Figure S2 Quantification of (A) *CTCF* and (B) *BORIS* transcripts in various tissues and species relative to *GAPDH* as determined by real-time PCR.
(0.47 MB EPS)

Table S1 Primers used in this study.
(0.19 MB DOC)

Table S2 Vertebrate homologues of *CTCF* and *BORIS*.
(0.11 MB DOC)
