# Supplementary material for: The Evolution of Epigenetic Regulators CTCF and BORIS/CTCFL in Amniotes
Source: PLoS Genet. 2008 Aug 29;4(8):e1000169. doi: 10.1371/journal.pgen.1000169 (PMC2515639; doi:10.1371/journal.pgen.1000169)
Supplement: Table S1 — Primers used in this study. (0.19 MB DOC) [file pgen.1000169.s003.doc]

**Table S1.** Primers used in this study.

| **Species** | **Gene** | **Primer Name** | **Sequence** | **Product size** | **Location** | **Description** |
| --- | --- | --- | --- | --- | --- | --- |
| Cattle | CTCF | btCTCF_F1 | TACAGATGGTGATGATGGAACAGC | 737 bp | Exons 1-2 | RT-PCR primers |
|  |  | btCTCF_R1 | GAATGCCCTGCCACAGAGATG |  |  |  |
|  |  | btCTCF_qF1 | GCATCGTCGTTACAAACACACC | 390 bp | Exons 3-6 | qPCR primers* |
|  |  | btCTCF_qR1 | CATTTCTTGCCCTGCTCAAT |  |  |  |
|  | BORIS | btBORIS_F8 | CACGCTTCTTGGGTGAGGAC | 208 bp | 5’UTR-exon 1 | Used to amplify EU527855 |
|  |  | btBORIS_R8 | CTCTGGTTCTTTGATTTGGGTG |  |  |  |
|  |  | btBORIS_F4 | CATAATGGCAGGGGCTGAG | 1069 bp | Exons 1-6 |  |
|  |  | btBORIS_R4 | GCAGAGGGAACACTGAAAGG |  |  |  |
|  |  | btBORIS_F5 | GCCACATCCGCTCACACAC | 892 bp | Exons 6-11 |  |
|  |  | btBORIS_R5 | CCTGCCTCCTGTCACTTATTCATC |  |  |  |
|  |  | btBORIS_F6 | AAAAGAAAGAGCAACGAAGAGGAA | 1509 bp | Exons 11-3’UTR |  |
|  |  | btBORIS_R6 | TATGGGAGGGGAAGAGTTCACAG |  |  |  |
|  |  | btBORIS_F2 | GCACTCCAAGAAACAGCACAATA | 242 bp | Exons 8-10 | RT-PCR and qPCR primers |
|  |  | btBORIS_R2 | GCTCTCCACTCACCAGGGATAC |  |  |  |
|  |  | btBORIS_F1 | GGACCCTTCCAGACCACGAT | 891 bp | Exons 1-5 | Extra RT-PCR primers |
|  |  | btBORIS_R4 | GCAGAGGGAACACTGAAAGG |  |  |  |
|  | GAPDH | btGAPDH_qF1 | GTGATGCTGGTGCTGAGTATGTG | 299 bp | Exons 4-7 | qPCR control |
|  |  | btGAPDH_qR1 | AGTCTTCTGGGTGGCAGTGATG |  |  |  |
| Wallaby | CTCF | meCTCF_F1 | ATGGAAGGTGAGGCAGTTGAAG | 2214 bp | N-term to C-term | Used to amplify EU527852 |
|  |  | meCTCF_R1 | CCAGTTTGGTGGCAGAGC |  |  |  |
|  |  | meCTCF_F3 | CCAGCCAGCCCAAGCAAG | 1524 bp | C-term to 3’UTR |  |
|  |  | meCTCF_R5 | TCCTGAACACTTTGCTGCTTTCTT |  |  |  |
|  |  | meCTCF_R_F1 | TTTATTCCGAGCACATGGATACTGG | 99 bp | 3'UTR | Used as nested forward primers for 3’RACE |
|  |  | meCTCF_R_F2 | GGTTTGGAAGCTGGGAAGGTGAA |  |  |  |
|  |  | oCTCF_R1 | GCAAGGCAAGAAATGTCGTTATTG | - | Exon 3 | Sequencing primer |
|  |  | meCTCF_F12 | GGGGCTTACGAGAATGAGGT | 547 bp | Exons 1-2 | RT-PCR primers |
|  |  | meCTCF_R11 | CTGTGTGAGTATTTAGGTGGTTCC |  |  |  |
|  |  | meCTCF_F11 | CGTTTCCGTGTATGACTTTGAG | 287 bp | Exons 1-2 | qPCR primers |
|  |  | meCTCF_R11 | CTGTGTGAGTATTTAGGTGGTTCC |  |  |  |
|  | BORIS | meBORIS_F19 | GGAATGGGGACGGAGGC | 1428 bp | N-term to C-term | Used to amplify EU527856 |
|  |  | meBORIS_R9 | GCAAGAAAGGCACGTAAATGGT |  |  |  |
|  |  | meBORIS_F4 | ATGCGTTCACATACTGGAGA | 2097 bp | ZF to 3’UTR |  |
|  |  | meBORIS_R5 | AGGCATTTCATTTGAGTTATTAGA |  |  |  |
|  |  | meBORIS_F8 | ACGAGATGGAGCTGGTGGAGA | - | N-term | Sequencing primer |
|  |  | meBORIS_R8 | CATGGCAGTGTCGGCTT | - | 3' UTR | Sequencing primer |
|  |  | meBORIS_F17 | GAAACCATTTACGTGCCTTTCTT | 284 bp | Exons 8-10 | RT-PCR and qPCR primers |
|  |  | meBORIS_R17 | CTGGCATCTGCTCAACAACTTCT |  |  |  |
|  |  | oBORIS_F1 | TTATGAATGCTACGTCTGCCATGC | 355 bp | Exons 6-8 | Extra RT-PCR primers |
|  |  | oBORIS_R1 | GGTGAATGGTTTCTCTCCTGTGTG |  |  |  |
|  | GAPDH | meuGAPDH_qF1 | AAGTTCAAGGGCACTGTCAAGG | 131 bp | Exons 2-3 | qPCR control |
|  |  | meuGAPDH_qR1 | GACTCTACAACATACTCGGCTCCA |  |  |  |
| Platypus | CTCF | meCTCF_F1 | ATGGAAGGTGAGGCAGTTGAAG | 1143 bp | N-term to ZF | Used to amplify EU527853 |
|  |  | oaCTCF_R_R2 | GCACTGGAATGGACGCTCT |  |  |  |
|  |  | oaCTCF_F3 | AAACGTCATATTCGCTCTCACAC | 686 bp | ZF |  |
|  |  | oaCTCF_R3 | TTACCCTTCTTGGTTTCTCCTC |  |  |  |
|  |  | oaCTCF_F4 | TGGTGTAGAAGGAGAGAATGGAGGA | 1454 bp | ZF to 3’UTR |  |
|  |  | oaCTCF_R4 | CAGAGCAAAGAAAGTGTAGGTGTGAA |  |  |  |
|  |  | oaCTCF_F7 | CTTCTACGTCATCCTCCCAAG | 1007 bp | 3’UTR |  |
|  |  | oaCTCF_R7 | TGTTAATCCGTTATTATTTATTAGCTG |  |  |  |
|  |  | oaCTCF_R_F1 | ATGACTTCTATGGTGAAAGCAAAGTGG | 93 bp | 3’UTR | Used as nested forward primers for 3’RACE |
|  |  | oaCTCF_R_F2 | GGGTTTGAAAGCTAGGAAGGAGAATA |  |  |  |
|  |  | oaCTCF_F12 | TCAGGAAGCAGAGGCAACC | 638 bp | N-term to ZF | RT-PCR primers |
|  |  | oaCTCF_R12 | ATTTGTGTGGTCTTTCATCAGTGT |  |  |  |
|  |  | oaCTCF_F11 | CAATGGCGAGGTGGAGAC | 375 bp | N-term to ZF | qPCR primers |
|  |  | oaCTCF_R12 | ATTTGTGTGGTCTTTCATCAGTGT |  |  |  |
|  | BORIS | oaBORIS_F1 | GATCCCGGCAGAAGGAAAC | 381 bp | Exons 1-3 | Used to amplify EU527857 |
|  |  | oaBORIS_R7 | GTGGCGATTGAGACTTGACTG |  |  |  |
|  |  | oaBORIS_F2 | CCACAGGATATGTCTCCGTCAGT | 1179 bp | Exons 2-10 |  |
|  |  | oaBORIS_R2 | TTGGCTTGTACCGTGCTCTGATTT |  |  |  |
|  |  | oaBORIS_R_F3 | CGGTTGGAGATACGTCCGAGATGAAA | 333 bp | 3' UTR |  |
|  |  | oaBORIS_R_F4 | CACATGAAGTGATACTCAGCCAGATGGA |  |  |  |
|  |  | oaBORIS_F10 | ACCCTTTTCCTGCCTTCACTGC | 291 bp | Exons 8-10 | RT-PCR and qPCR primers |
|  |  | oaBORIS_R10 | GTATCTCCAACCGATGTCTGCGTA |  |  |  |
|  |  | oaBORIS_F7 | TATGGCAGAAGAAGGAAAGCAC | 276 bp | Exons 2-3 | Extra RT-PCR primers |
|  |  | oaBORIS_R7 | GTGGCGATTGAGACTTGACTG |  |  |  |
|  | GAPDH | oanGAPDH_qF1 | GTATGATTCCACCCACGGCA | 210 bp | Exons 3-5 | qPCR control |
|  |  | oanGAPDH_qR1 | CGCTTGGCTCCTCCCTTC |  |  |  |
| Bearded Dragon | CTCF | acaCTCF_F1 | GTGACATGGAGGGCGAAGTAGTT | 958 bp | N-term to ZF | Used to amplify EU527854 |
|  |  | acaCTCF_R5 | CCTGTATGAGTGTTGAGATGGTTTC |  |  |  |
|  |  | acaCTCF_F2 | AAAGACATTCCAGTGTGAACTGTG | 2762 bp | ZF to 3’UTR |  |
|  |  | acaCTCF_R1 | CAGCAGTATATTCTCCTTCCCAG |  |  |  |
|  |  | pvCTCF_F1 | CTGCCTTTGTCTGCTCCAAGTGT | - | ZF | Sequencing primer |
|  |  | pvCTCF_R1 | GCAAAGTATCAGGGAAGAAAGACACC | - | 3' UTR | Sequencing primer |
|  |  | pviCTCF_F1 | ATGGCGAAGTGGAGACATTAGA | 438 bp | Exons 1-2 | RT-PCR primers |
|  |  | pviCTCF_R1 | CTGTGTGAGTGTTGAGGTGATTT |  |  |  |
|  |  | pviCTCF_qF1 | GGTGAGTTGGTTCGGCATC | 187 bp | Exons 3-4 | qPCR primers |
|  |  | pviCTCF_qR1 | GCCTCTTCAGTTTGTAAGTGTCTCT |  |  |  |
|  | BORIS | acaBORIS_F1 | AGGCTTTGGGAGAAGGAGAGAAAC | 859 bp | N-term to ZF | Used to amplify EU527858 |
|  |  | pvBORIS_R3 | CCTGTATGGGAGCGAATGTGA |  |  |  |
|  |  | acaBORIS_F5 | GAATGTGATATGGCCTTTGTGAC | 787 bp | ZF to C-term |  |
|  |  | pviBORIS_R3 | CTTTGCTGGGCTGAATCGCT |  |  |  |
|  |  | pviBORIS_R_F1 | CAAACAGGAACGCCACATGGTGATA | 624 bp | ZF | Forward primer for 3’ RACE |
|  |  | pviBORIS_F3 | CAGGAACGCCACATGGTGATA | 247 bp | Last two exons | RT-PCR and qPCR primers |
|  |  | pviBORIS_R3 | CTTTGCTGGGCTGAATCGCT |  |  |  |
|  |  | pviBORIS_F1 | CGTCACATTCGCTCCCATAC | 299 bp | ZF | Extra RT-PCR primers (ZF) |
|  |  | pviBORIS_R1 | GCACCTCAACGGCACTTCT |  |  |  |
|  | GAPDH | pviGAPDH_qF1 | GTGGAGGGATGGCAGAGGT | 130 bp | Exons 6-7 | qPCR control |
|  |  | pviGAPDH_qR1 | TGGAGTTGGGACACGGAAAG |  |  |  |

*qPCR primers are those primers used in ‘quantitative’ or real-time PCR.
