# Supplementary material for: The Evolution of Epigenetic Regulators CTCF and BORIS/CTCFL in Amniotes
Source: PLoS Genet. 2008 Aug 29;4(8):e1000169. doi: 10.1371/journal.pgen.1000169 (PMC2515639; doi:10.1371/journal.pgen.1000169)
Supplement: Table S2 — Vertebrate homologues of CTCF and BORIS. (0.11 MB DOC) [file pgen.1000169.s004.doc]

**Table S2.** Vertebrate homologues of *CTCF* and *BORIS*.

| **Common Name** | **Species Name** | **Classification** | **Gene** | **Accession number** | **Experimental evidence?** | Reference |
| --- | --- | --- | --- | --- | --- | --- |
| Human | Homo sapiens | Eutherian mammal | hsaBORIS | NM_080618.2 | Yes | [1] |
| Human | Homo sapiens | Eutherian mammal | hsaCTCF | NM_006565.2 | Yes | [2] |
| Chimpanzee | Pan troglodytes | Eutherian mammal | ptrBORIS | ENSPTRP00000023498^**^ | No |  |
| Chimpanzee | Pan troglodytes | Eutherian mammal | ptrCTCF | XM_511035.2 | No |  |
| Rhesus macaque | Macaca mulatta | Eutherian mammal | rheBORIS | ENSMMUP00000022088^**^ | No |  |
| Small-eared galago | Otolemur garnettii | Eutherian mammal | ogaBORIS | ENSOGAP00000013137^**^ | No |  |
| Mouse | Mus musculus | Eutherian mammal | mmuBORIS | NM_001081387.2 | Yes | [1] |
| Mouse | Mus musculus | Eutherian mammal | mmuCTCF | NM_007794.1 | Yes | [1] |
| Rat | Rattus norvegicus | Eutherian mammal | rnoBORIS | ENSRNOP00000034635^**^ | No |  |
| Rat | Rattus norvegicus | Eutherian mammal | rnoCTCF | NM_031824.1 | Yes |  |
| Cattle | Bos taurus | Eutherian mammal | btaBORIS | EU527855 | Yes | This Study |
| Cattle | Bos taurus | Eutherian mammal | btaCTCF | NM_001075748.1 | Yes |  |
| Dog | Canis familiaris | Eutherian mammal | cfaBORIS | XM_534463 with edition | No |  |
| Dog | Canis familiaris | Eutherian mammal | cfaCTCF | XM_859339.1 | No |  |
| Western European hedgehog | Erinaceus europaeus | Eutherian mammal | eeuBORIS | ENSEEUP00000010716^**^ | No |  |
| European shrew | Sorex araneus | Eutherian mammal | sarCTCF | ENSSARP00000005634^**^ | No |  |
| Horse | Equus caballus | Eutherian mammal | ecaBORIS | GENSCAN00000098721^**^ | No |  |
| Horse | Equus caballus | Eutherian mammal | ecaCTCF | XM_001497850 with edition | No |  |
| Pig | Sus scrofa | Eutherian mammal | sscBORIS | NM_001110174.1 | No |  |
| African savanna elephant | Loxodonta africana | Eutherian mammal | lafBORIS | ENSLAFP00000007760^**^ | No |  |
| African savanna elephant | Loxodonta africana | Eutherian mammal | lafCTCF | ENSLAFT00000007101^**^ with trace archive data | No |  |
| Nine-banded armadillo | Dasypus novemcinctus | Eutherian mammal | dnoBORIS | ENSDNOP00000012186^**^ | No |  |
| Gray, short-tailed opossum | Monodelphis domestica | Marsupial mammal | mdoBORIS | ENSMODP00000020611^**^ | No |  |
| Gray, short-tailed opossum | Monodelphis domestica | Marsupial mammal | mdoCTCF | ENSMODP00000007129^**^ | No |  |
| Tammar wallaby | Macropus eugenii | Marsupial mammal | meuBORIS | EU527856 | Yes | This Study |
| Tammar wallaby | Macropus eugenii | Marsupial mammal | meuCTCF | EU527852 | Yes | This Study |
| Duck-billed platypus | Ornithorhynchus anatinus | Monotreme mammal | oanBORIS | EU527857 | Yes | This Study |
| Duck-billed platypus | Ornithorhynchus anatinus | Monotreme mammal | oanCTCF | EU527853 | Yes | This Study |
| Green Anole | Anolis carolinensis | Squamate Reptile | acaBORIS^†^ | Scaffold_190^**^, with genome scan | No |  |
| Green Anole | Anolis carolinensis | Squamate Reptile | acaCTCF | Scaffold_448^**^, with genome scan | No |  |
| Central bearded dragon | Pogona vitticeps | Squamate Reptile | pviBORIS^†^ | EU527858 | Yes | This Study |
| Central bearded dragon | Pogona vitticeps | Squamate Reptile | pviCTCF | EU527854 | Yes | This Study |
| Chicken | Gallus gallus | Neognathae bird | ggaCTCF | NM_205332.4 | Yes | [3] |
| Zebra finch | Taeniopygia guttata | Neognathae bird | tguCTCF | Derived from EST CK317499 and trace data | Partial |  |
| African clawed frog | Xenopus laevis | Amphibian | xlaCTCF | NM_001086461.1 | Yes | [4] |
| Western clawed frog | Xenopus tropicalis | Amphibian | xtrCTCF | ENSXETP00000034066^**^ | No |  |
| Zebrafish | Danio rerio | Ray finned fish | dreCTCF | NM_001001844.1 | Yes | [5] |
| Green spotted puffer | Tetraodon nigroviridis | Ray finned fish | tniCTCF | GSTENT00017695001^**^ | No |  |
| Tiger puffer | Takifugu rubripes | Ray finned fish | truCTCF | NEWSINFRUT00000156554^**^ | No |  |
| Three-spined stickleback | Gasterosteus aculeatus | Ray finned fish | gacCTCF_2 | ENSGACT00000003281^**^ | No |  |
| Japanese medaka | Oryzias latipes | Ray finned fish | olaCTCF_1 | ENSORLT00000011018^**^ | No |  |
| Three-spined stickleback | Gasterosteus aculeatus | Ray finned fish | gacCTCF_1 | ENSGACT00000020981^**^ | No |  |
| Japanese medaka | Oryzias latipes | Ray finned fish | olaCTCF_2 | ENSORLT00000022987^**^ | No |  |
| Sea lamprey | Petromyzon marinus | Jawless fish | pmaCTCF | Derived from EST DW022714 and GENSCAN00000118609^**^ | Partial |  |

^†^When 3’ RACE was performed on bearded dragon *BORIS* cDNA (EU527858) we could find no sequence homologous to the last coding exon of human *BORIS,* and discovered by genomic PCR that the 3’UTR follows on directly from the region homologous to the second-last exon of human *BORIS* without an interspersed intron (data not shown). Nor could we detect sequence homologous to this region in green anole, so we predict that reptilian *BORIS* is missing this exon entirely.

^**^Indicates which sequences can be retrieved from Ensembl ([http://www.ensembl.org](http://www.ensembl.org/)). All other sequences can be found at NCBI ([http://www.ncbi.nlm.nih.gov](http://www.ncbi.nlm.nih.gov/)).

### References

1. Loukinov DI, Pugacheva E, Vatolin S, Pack SD, Moon H, et al. (2002) BORIS, a novel male germ-line-specific protein associated with epigenetic reprogramming events, shares the same 11-zinc-finger domain with CTCF, the insulator protein involved in reading imprinting marks in the soma. Proc Natl Acad Sci U S A 99: 6806-6811.

2. Filippova GN, Fagerlie S, Klenova EM, Myers C, Dehner Y, et al. (1996) An exceptionally conserved transcriptional repressor, CTCF, employs different combinations of zinc fingers to bind diverged promoter sequences of avian and mammalian c-myc oncogenes. Mol Cell Biol 16: 2802-2813.

3. Klenova EM, Nicolas RH, Paterson HF, Carne AF, Heath CM, et al. (1993) CTCF, a conserved nuclear factor required for optimal transcriptional activity of the chicken c-myc gene, is an 11-Zn-finger protein differentially expressed in multiple forms. Mol Cell Biol 13: 7612-7624.

4. Burke LJ, Hollemann T, Pieler T, Renkawitz R (2002) Molecular cloning and expression of the chromatin insulator protein CTCF in Xenopus laevis. Mech Dev 113: 95-98.

5. Pugacheva EM, Kwon YW, Hukriede NA, Pack S, Flanagan PT, et al. (2006) Cloning and characterization of zebrafish CTCF: Developmental expression patterns, regulation of the promoter region, and evolutionary aspects of gene organization. Gene 375: 26-36.
